# Mitigation of paclitaxel-induced peripheral neuropathy in breast cancer patients using limb-cooling apparatus: a study protocol for a randomized controlled trial

**DOI:** 10.3389/fonc.2023.1216813

**Published:** 2023-07-07

**Authors:** Chikako Funasaka, Akiko Hanai, Sadamoto Zenda, Keita Mori, Makoto Fukui, Nami Hirano, Rie Shinohara, Nozomu Fuse, Masashi Wakabayashi, Mai Itagaki, Yutaka Tomioka, Michihiko Nishina, Yasuaki Arai, Takahiro Kogawa, Yukinori Ozaki, Meiko Nishimura, Takayuki Kobayashi, Fumikata Hara, Toshimi Takano, Toru Mukohara

**Affiliations:** ^1^ Department of Medical Oncology, National Cancer Center Hospital East, Kashiwa, Japan; ^2^ Department of Experimental Therapeutics, National Cancer Center Hospital East, Kashiwa, Japan; ^3^ Medical Data Mathematical Reasoning Team, Advanced Data Science Project, RIKEN, Yokohama, Japan; ^4^ Department of Radiation Oncology, National Cancer Center Hospital East, Kashiwa, Japan; ^5^ Supportive and Palliative Care Research Support Office, National Cancer Center Hospital East, Kashiwa, Japan; ^6^ Department of Biostatistics, Clinical Research Center, Shizuoka Cancer Center, Shizuoka, Japan; ^7^ Clinical Research Support Office, National Cancer Center Hospital East, Kashiwa, Japan; ^8^ Section of Research Administration, National Cancer Center Hospital East, Kashiwa, Japan; ^9^ Division of Medical Device Innovation, National Cancer Center Hospital East, Kashiwa, Japan; ^10^ Planning and Product Development Division, Nippon Sigmax Co, Ltd., Shinjuku, Tokyo, Japan; ^11^ Department of Diagnostic Radiology, National Cancer Center Hospital, Tokyo, Japan; ^12^ Department of Advanced Medical Development, Cancer Institute Hospital of Japanese Foundation for Cancer Research, Tokyo, Japan; ^13^ Department of Breast Medical Oncology, Cancer Institute Hospital of Japanese Foundation for Cancer Research, Tokyo, Japan

**Keywords:** breast cancer, paclitaxel, chemotherapy induced peripheral neuropathy, limb cooling therapy, circulation cooling system

## Abstract

**Background:**

Chemotherapy-induced peripheral neuropathy (CIPN) is one of the most common adverse events that can significantly impair the quality of life of patients. Although limb cooling may be beneficial for preventing CIPN, logistical challenges exist in ensuring consistent efficacy and safety. The purpose of this randomized controlled trial is to validate whether limb cooling with strict temperature control can reduce CIPN in patients with breast cancer receiving weekly paclitaxel as a perioperative treatment.

**Methods:**

This study is a multicenter, double-blinded, randomized controlled trial. We plan to enroll patients with breast cancer who are scheduled to receive 12 weekly doses of paclitaxel (60 min 80 mg/m^2^ intravenous infusion) as perioperative chemotherapy. Patients will be randomly divided into the intervention or control groups and undergo limb cooling therapy maintained at a constant temperature of 13°C and 25°C, respectively. The primary endpoint is the proportion of patients who report Patient Neurotoxicity Questionnaire (PNQ) ≥ D in their limbs by the end of the study treatment or at the time of discontinuation.

**Discussion:**

The results of this trial will contribute to the establishment of new evidence for limb cooling therapy in the mitigation of CIPN and present a safe and stable cooling device that may be suitable for use in the clinic.

**Clinical trial registration:**

https://jrct.niph.go.jp/en-latest-detail/jRCT2032210115, identifier jRCT2032210115.

## 1 Introduction

Chemotherapy-induced peripheral neuropathy (CIPN) is a common adverse event that can significantly impair the quality of life (QOL) of patients. While standard chemotherapy often uses platinum-based drugs, such as cisplatin and oxaliplatin, and taxane-based drugs, such as paclitaxel (PTX), the side effects of these drugs include CIPN, limb numbness, and pain (1). In a retrospective cohort study of 488 female patients receiving docetaxel or paclitaxel, a dose-limiting event occurred in 120 (24.6%) patients, and more than one-third (37.3%) of these events were attributed to CIPN (1). CIPN can persist for several months or years and negatively impacts various aspects of daily life, including occupational activities and sleep (2). In a Cancer Survivors’ Concerns and Burdens Report issued in 2013, which surveyed 4,054 cancer survivors across Japan, CIPN ranked third among the ‘symptoms, side effects and sequelae’ that burden cancer survivors, after hair loss and other anti-cancer drug side effects and was the leading chemotherapy-related cause of poor QOL (3).

The dose-dependent nature of CIPN, with more severity and frequency observed at higher doses, may necessitate dose reduction or discontinuation of treatment to resolve CIPN symptoms. Although PTX is administered at 80 mg/m^2^ every week for 12 weeks as part of the standard perioperative chemotherapy for breast cancer, 73.4% of patients have reported wanting to reduce or stop treatment because of CIPN (2). However, a reduction in dose intensity (mg/m^2^/week) is known to negatively affect disease-free survival. Furthermore, breast cancer survivors with CIPN have significantly higher 5-year survival rates than those without CIPN (4). These findings suggest that it is critical to maintain dosing schedules as a reduction in dose intensity owing to CIPN may lead to poorer treatment outcomes. Therefore, preventing CIPN during perioperative chemotherapy for breast cancer could improve both QOL and treatment outcomes.

Currently, the most promising method for mitigating CIPN is limb cooling, also known as cryotherapy, which aims to reduce blood flow to the limbs and prevent the distribution of chemotherapy drugs to the peripheral nervous system (5). Most current evidence available combines limb cooling with taxane-based therapy as this regime received a level of evidence of II and a grade of recommendation of C in the European Society for Medical Oncology guidelines (6). In addition, the American Society of Clinical Oncology guidelines indicate that limb cooling therapy and compression therapy may prevent CIPN symptoms but make no recommendation for the utilization of these interventions outside the context of clinical trials (7). An observational study showed that the incidence of CIPN was lower in a group of patients who used Elasto-Gel^®^ gloves and socks when receiving docetaxel [odds ratio (OR) = 0.60, 95% CI = 0.42–0.86] (5). Hanai et al. conducted a prospective self-controlled trial to evaluate the efficacy of limb cooling for CIPN. The results showed that tactile function was maintained significantly better on the side on which patients wore the cooling glove/sock (dominant limb side) than on the side on which patients did not wear the cooling glove/sock, suggesting a CIPN-reducing effect of the intervention (8). In a randomized trial, the primary endpoint of a greater than 10% or 6-point reduction in the Functional Assessment of Cancer Therapy-Neurotoxicity score was significantly improved in patients who used the Elasto-Gel^®^ cooling glove/sock compared to patients who did not (9). Importantly, the safety of cooling gloves and socks has yet to be adequately evaluated. In a larger randomized controlled trial evaluating the efficacy of frozen gloves in preventing CIPN with oxaliplatin, docetaxel, or paclitaxel, the patients that used frozen gloves demonstrated no improvement in CIPN subscales, and one-third of the patients discontinued the study before the end of treatment because of discomfort (10). Similarly, several other trials using cooling gloves/socks reported high withdrawal rates among patients because of cooling discomfort (5, 10). The Food and Drug Administration issued a Class II Recall of Elasto-Gel^®^ in the United States owing to two cases of frostbite in off-label use (11). A global survey on the use of cryotherapy indicated that barriers to implementation include insufficient evidence of efficacy, logistics, and patient discomfort (12). Therefore, the development of medical cooling devices that are safe and efficient for patients is urgently required.

To address this unmet medical need, we designed the following double-blinded, inter-individual, randomized controlled trial to validate the use of a cooling device with strict temperature control in mitigating CIPN in patients with breast cancer undergoing weekly PTX treatment: a randomized Control trial to Evaluate the mitigation of CIpn by Limb-cooling Apparatus (CECILIA).

## 2 Methods and analysis

### 2.1 Participants, interventions, and outcomes

#### 2.1.1 Study setting

**Figure 1:**
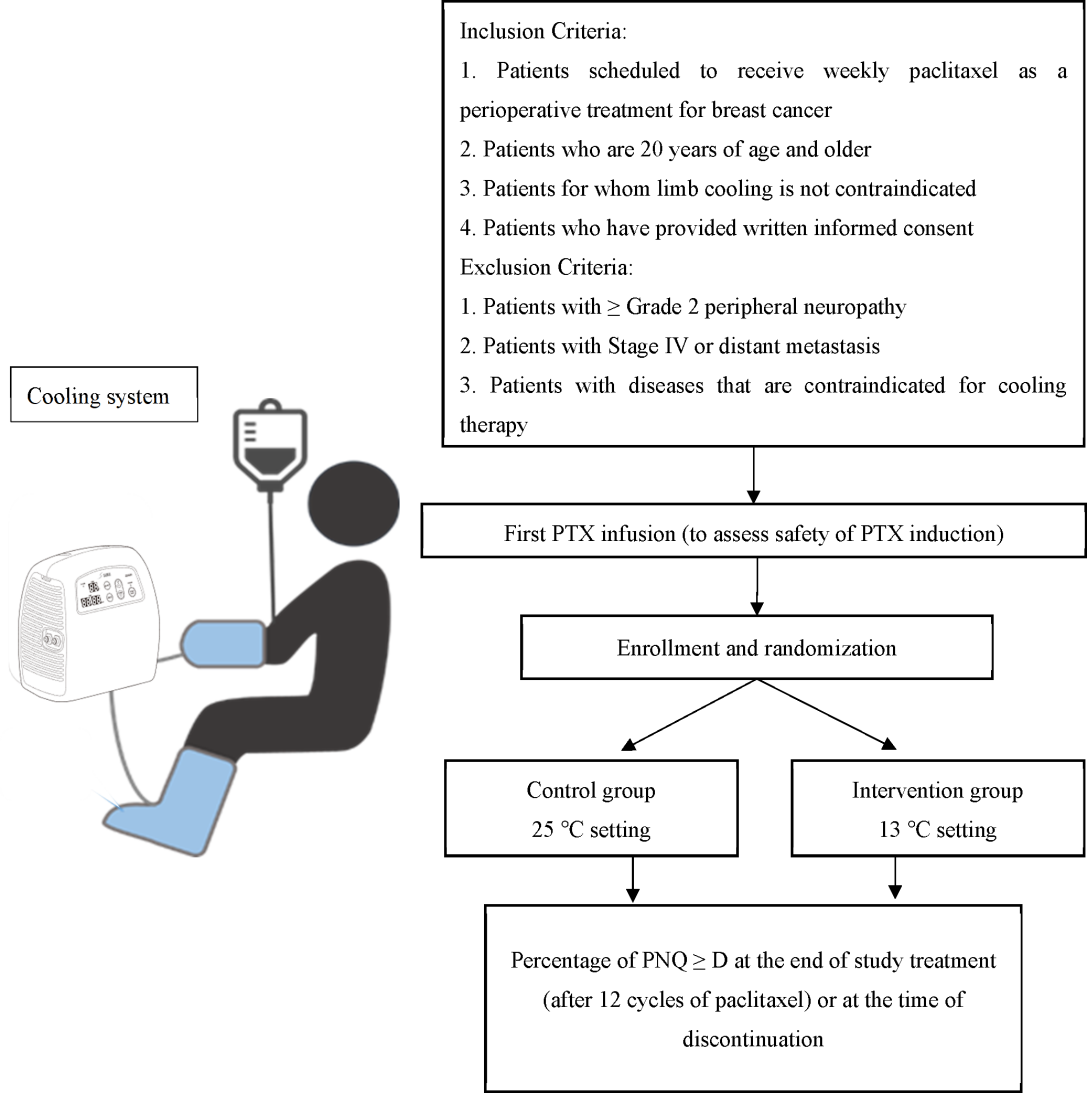
Schematic overview of the CECILIA trial.

This multicenter, double-blinded, randomized, controlled trial will be conducted at two participating institutions: the National Cancer Center Hospital East and the Japanese Foundation for Cancer Research. A schematic overview of the trial is shown in **Figure 1**.

#### 2.1.2 Study eligibility

Inclusion criteria are as follows: 1) patients with clinical stage I-III breast cancer, including new primary breast cancer and local recurrence breast cancer, scheduled to receive 12 weekly doses of PTX (80 mg/m^2^, 60 min intravenous infusion) with or without pertuzumab and/or trastuzumab as perioperative (including both pre- and post-operative) treatment; 2) patients ≥ 20 years of age by the date of enrollment; 3) a performance status of 0 or 1 according to the Eastern Cooperative Oncology Group; 4) written consent to participate in the trial; 5) adequate organ function, as confirmed by clinical laboratory tests within 14 days of the first dose of PTX [neutrophil count ≥ 1,000/mm^3^, platelet count ≥ 75,000/mm^3^, hemoglobin ≥ 8.0 g/dL, serum creatinine ≤ 2.0 mg/dL or calculated (Cockcroft–Gault formula) creatinine clearance ≥ 50 mL/min; total bilirubin ≤ 1.5 mg/dL; alanine and aspartate aminotransferase ≤ 100 U/L]; and 6) patients permitted to receive a second PTX dose based on their response to the first dose.

Exclusion criteria are as follows: 1) patients with Common Terminology Criteria for Adverse Events (CTCAE) v5.0 Grade ≥ 2 ‘peripheral sensory neuropathy’ or Grade ≥ 1 ‘edema limbs’ in any of the extremities at enrollment; 2) patients with missing fingers or toes; 3) other peripheral complications, such as cold ganglionopathy, cold urticaria, Raynaud’s symptoms, peripheral arterial ischemic symptoms, and hand-foot syndrome; 4) history of varicose veins or varicose vein thrombosis; 5) history of coagulation disorders, such as antiphospholipid antibody syndrome, protein C/S deficiency, and antithrombin deficiency; 6) planned administration of duloxetine or other neuropathic drugs during the study period (the applicability of drugs other than duloxetine will be determined by the study secretariat on an individual basis); 7) severe alcohol hypersensitivity; 8) patients who received any previous treatment for cancer within 7 days of the first dose of PTX, including chemotherapy, molecular targeted therapy, antibody therapy (with the exception of trastuzumab and pertuzumab), hormonal therapy, immunotherapy, and radiotherapy; 9) patients who have not recovered from toxicity due to previous therapy to grade 1 or less or to baseline according to CTCAE v5.0, except for abnormal blood tests to the extent inclusion criteria 5 are met or events with stable symptoms such as grade 2 alopecia and Grade 2 skin hyperpigmentation; 10) history of trauma or surgery to the fingers and toes within 1 month of enrollment; 11) comorbidities, such as poorly controlled diabetes mellitus, autoimmune neuropathy (e.g., Guillain-Barre syndrome, Fisher syndrome, and chronic inflammatory demyelinating polyneuropathy), and cerebrovascular disease with incomplete or complete paralysis; 12) patients who had undergone major surgery within 4 weeks of enrollment. Breast cancer surgical interventions (total mastectomy, partial mastectomy, and axillary lymph node dissection) or central vein catheter port placement were allowed unless they were not performed within 2 weeks of enrollment; and 13) the investigator or sub-investigator determines the patient unsuitable for enrollment in the trial.

#### 2.1.3 Treatment methods

**Figure 2:**
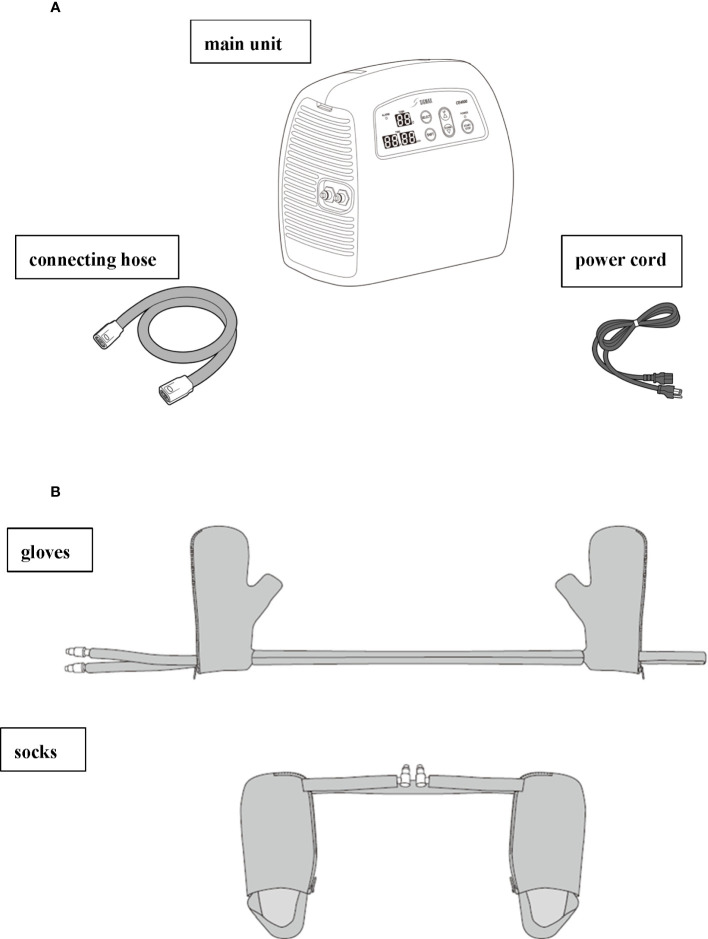
Illustration of the Icing System (CE4000) apparatus provided by Nippon Sigmax. **(A)** The parts of the CE4000 cooling system, including the main unit, cord, and power cord. **(B)** The gloves (right) and socks (left) to be worn by patients 15 min before the treatment is initiated, during the 60 min PTX treatment, and until 15 min after the treatment is completed.

Patients will receive limb cooling therapy at 13°C and 25°C for the intervention group and control group, respectively. The intervention will last for a total of 90 min, initiated 15 min before until 15 min after the 60 min weekly PTX administration (cycles 2–12). The temperature of the cooling system (Icing System, CE4000; Nippon Sigmax Co., Ltd., Tokyo, Japan) will be pre-set at 13°C and 25°C, and the temperature monitor on the apparatus will not be visible to ensure double-blindness. An illustration of the cooling system setup is shown in **Figure 2**.

#### 2.1.4 Endpoints

The primary endpoint is the proportion of patients who report Patient Neurotoxicity Questionnaire (PNQ) grade ≥ D in their limbs by the end of the study treatment or at the time of discontinuation (13). There are eleven secondary outcomes, defined as the proportion of patients who 1) report Grade ≥ 2 ‘peripheral motor neuropathy’ or ‘peripheral sensory neuropathy,” according to CTCAE version 5.0; 2) report ‘numbness or tingling’ in limbs ≥ ‘moderate’ severity and ‘somewhat’ inference, based on the National Cancer Institute (NCI)-Patient Reported Outcomes (PRO)-CTCAE; 3) report ‘pain’ ≥ ‘sometimes’ frequency, ‘moderate’ severity, and ‘somewhat’ inference, according to the NCI-PRO-CTCAE; 4) report PNQ ≥ D at 3 months after the end of PTX treatment and at the end of the follow-up period; 5) report subjective symptoms of CIPN as measured using the European Organization for Research and Treatment of Cancer Quality of Life Questionnaire (EORTC QLQ) C-30 and EORTC QLQ-CIPN20; 6) develop other adverse events measured by CTCAE v.5.0; 7) develop frostbite according to the Japanese Dermatological Association (14); and 8) complete limb cooling until the 12^th^ cycle of PTX. Lastly, the 9) skin temperature of the hand and foot before and during cold exposure; 10) relative dose intensity of PTX; and 11) incidence of mechanical issues of the cooling device.

#### 2.1.5 Follow-up

The follow-up visit for safety evaluation for all enrolled patients will take place within 30 days (± 28 days) after the last study intervention and before the start of any follow-up treatments, including surgery. The follow-up visits will continue every 3 months (± 1 month) from the end of cycle 12 of PTX or the date of discontinuation of the study treatment until the end of the planned observation period, and every 6 months (± 1 month) thereafter. Follow-up can be terminated for patients who no longer experience numbness or pain in their limbs, or for whom post-treatment that could cause peripheral neuropathy is initiated. The planned enrollment period is 2 years (October 2021 to September 2023), and the planned observation period is 3 months from when the last patient is enrolled.

#### 2.1.6 Sample size

To confirm the effectiveness of limb cooling, the sample size was calculated based on the results of previous studies whereby the proportion of PNQ ≥ D was 15% in the limb cooling intervention group and 37% in the control group. Therefore, we determined that the required number of patients with 2.5% one-sided significance level and 80% power in the Fisher’s exact test was 138. Assuming a small number of dropouts, the planned total number of patients was set to 150 (75 patients per group). If the one-sided p-value of Fisher’s exact test in terms of the proportions of PNQ ≥ D is less than 2.5%, the limb cooling therapy at 13°C is the standard therapy for mitigation of chemotherapy-induced CIPN in patients with breast cancer who receive weekly PTX as perioperative treatment.

### 2.2 Assignment of interventions

#### 2.2.1 Randomization

Patients will be enrolled using a web-based registration system and randomly divided into intervention or control groups. The randomization results are not included in the enrollment confirmation email. Furthermore, investigators and site staff are prohibited from referencing the subjects’ allocation on the electronic data capture system. The temperature of the cooling equipment will be pre-set before the equipment is brought to the hospital, and the temperature will not be displayed on the equipment. Randomization will be performed using a minimization method to prevent the imbalance of the following components: (i) the presence or absence of Grade 1 peripheral neuropathy and (ii) institution (National Cancer Center Hospital East vs. Cancer Institute Hospital of Japanese Foundation for Cancer Research). Upon the completion of the study treatments, a questionnaire will be administered to the patients to determine which group they feel they have been allocated to. Additionally, a post-study review will be conducted to ensure that blindness has been maintained.

### 2.3 Data collection, management, and analysis

#### 2.3.1 Data collection

PNQs and NCI-PRO-CTCAE™ will be obtained after obtaining consent until the first cycle of PTX administration, at enrollment, on the day of PTX administration for cycles 3–12, at study discontinuation, and at the last follow-up period. CTCAE will be evaluated after obtaining consent until the first cycle of PTX administration, at enrollment, at the visit on the day of PTX administration for cycles 2–12, at study discontinuation, and during the last follow-up period. The EORTC QLQ C-30 and EORTC QLQ-CIPN20 will be administered at enrollment, on the day of PTX administration for cycles 3–12, and at study discontinuation.

#### 2.3.2 Statistical analysis

In terms of the primary endpoint, the proportion of PNQ ≥ D, a Fisher’s exact test will be performed for all randomized patients. If the one-sided p-value is ≤ 2.5%, the effectiveness of limb cooling with statistical significance will be confirmed. If PNQ ≥ D occurs, a descriptive summary will be provided using summary statistics such as median and interquartile range for the duration of PNQ ≥ D. If the safety of the intervention group is acceptable and the intervention group is superior to the control group for the other secondary endpoints, we will conclude that the intervention is a more useful treatment than the control treatment.

## 3 Discussion

The CECILIA study is the first multicenter, randomized, double-blinded trial investigating the effectiveness of limb cooling for mitigating PTX-induced peripheral neuropathy in patients with breast cancer using a circulation cooling system.

This study has several strengths. In a previous trial using frozen gloves/socks, the temperature of the gloves was difficult to control continuously, with gloves reaching an epidermal temperature ≤ 10°C immediately after wearing, which caused significant discomfort (8). However, in this study, we will use a circulating cooling system that maintains a stable temperature. Second, CECILIA utilizes the PNQ, a patient-reported outcome, as the primary endpoint. Although our proof-of-concept study suggested the effectiveness of limb cooling in reducing CIPN, the outcome was measured primarily using a tactile function test (8). Although this test was objective, it did not consider the true endpoint when measuring CIPN. The PNQ has an applicable degree of feasibility, validity, and usefulness for the diagnosis of CIPN and has been shown to be a more sensitive and compliant assessment tool than the Functional Assessment of Cancer Therapy/Gynecologic Oncology Group -Neurotoxicity and NCI-CTCAE scales when diagnosing and rating CIPN (15). Third, this is a double-blinded study. Although previous studies employed patient-reported outcomes as outcome measures, they were not blinded because of the nature of the products used (9, 10). Therefore, an unblind bias may have affected the results. In the CECILIA study, patients will be individually randomized, patients and investigators will be double-blinded, and the devices will be used at preset temperatures to minimize potential bias in this study.

While cryotherapy has been shown to be effective, concerns have been raised regarding its safety and tolerability (11, 12, 16). In this study, we will use a previously approved cooling device that can maintain a constant cool temperature to patients’ limbs. Currently, this device is widely used as a controlled medical device in Japan for the remission of pain in rheumatism, arthritis, and neuralgia, and for the control of bleeding, swelling, and pain due to trauma. To date, frostbite and other major complications have not been reported as adverse events of this intervention. To validate the safe application of this device in patients with breast cancer, we included safety as a secondary endpoint in this study. In studies investigating the efficacy of scalp cooling, switching from ice packs to circulatory cooling devices improved treatment results and safety (17, 18). Similarly, our circulatory medical device is expected to be more effective, safer, and more comfortable for patients than the conventional ice pack method as it can provide cooling at a stable temperature without relying on patients or medical staff for manual control.

In conclusion, we believe that the results of the CECILIA trial will provide new evidence to support the benefit of limb cooling therapy in the mitigation of CIPN, leading to the development of a safe cooling device for this purpose.

## Ethics statement

This study was approved by the National Cancer Center Institutional Review Board (clinical trial protocol number EPOC2002). The patients/participants provided their written informed consent to participate in this study.

## Author contributions

TM: project leader. CF, AH, SZ, MF, MW, NF, YT, KM, MiN, and TM: designing the protocol and planning the study. CF, AH, and TM: drafted the manuscript. All authors contributed to the article and approved the submitted version.
