# Supplementary material for: Mitigation of paclitaxel-induced peripheral neuropathy in breast cancer patients using limb-cooling apparatus: a study protocol for a randomized controlled trial
Source: Front Oncol. 2023 Jul 7;13:1216813. doi: 10.3389/fonc.2023.1216813 (PMC10361568; doi:10.3389/fonc.2023.1216813)
Supplement: Supplementary file 1 [file DataSheet_1.zip › PNQ english.pdf]

Patient Neurotoxicity Questionnaire (PNQ)©

Taxanes, Cisplatin and Carboplatin

Item 1.

☐ **A**	☐ **B**	☐ **C**	☐ **D***	☐ **E***
I have no numbness, pain or tingling in my hands or feet.	I have mild tingling, pain or numbness in my hands or feet. This does not interfere with my activities of daily living.	I have moderate tingling, pain or numbness in my hands or feet. This does not interfere with my activities of daily living.	I have moderate to severe tingling, pain or numbness in my hands or feet. This interferes with my activities of daily living.	I have severe tingling, pain or numbness in my hands or feet. It completely prevents me from doing most activities of daily living.

Item 2.

☐ **A**	☐ **B**	☐ **C**	☐ **D***	☐ **E***
I have no weakness in my arms or legs	I have a mild weakness in my arms or legs. This does not interfere with my activities of daily living.	I have moderate weakness in my arms or legs. This does not interfere of my activities of daily living.	I have moderate to severe weakness in my arms or legs. This interferes with my activities of daily living.	I have severe weakness in my arms or legs. It completely prevents me from doing most activities of daily living.

* Please indicate by placing an X in the box or writing in the space provided which activity or activities have been interfered with as a result of therapy.

My ability to:

☐ Button clothes	☐ Open doors	☐ Fasten buckles	☐ Write	☐ Sew
☐ Use a knife	☐ Put in or remove contact lenses	☐ Sleep	☐ Walk	☐ Work
☐ Use a fork	☐ Dial or use telephone	☐ Climb stairs	☐ Put on jewelry	☐ Tie shoes
☐ Use a spoon	☐ Operate a remote control	☐ Type on a keyboard	☐ Knit	☐ Drive
	☐ Other eating utensils, etc	☐ Perform activities of importance to me, specify: ______________________		
