# Supplementary material for: Mitigation of paclitaxel-induced peripheral neuropathy in breast cancer patients using limb-cooling apparatus: a study protocol for a randomized controlled trial
Source: Front Oncol. 2023 Jul 7;13:1216813. doi: 10.3389/fonc.2023.1216813 (PMC10361568; doi:10.3389/fonc.2023.1216813)
Supplement: Supplementary file 1 [file DataSheet_1.zip › pro-ctcae_japanese.pdf]

NCI- PRO-CTCAE™ ITEMS - JAPANESE

Item Library Version 1.0

As individuals go through treatment for their cancer they sometimes experience different symptoms and side effects. For each question, please select the one response that best describes your experiences over the past 7 days…

がんの治療を受けている方は、しばしば異なる症状や薬の副作用を経験いたします。それぞれの質問事項について、過去 7 日間にそれぞれの症状を経験されたかどうか、また経験された方は、その症状がどの程度だったか、もっとも自分の症状に適していると思われる回答を 1 つ選択してください。

**1. PRO-CTCAE™ Symptom Term:** Dry mouth				
口の中の乾き				
a. この７日の間で、口の中の乾きは一番ひどい時でどの程度でしたか？				
o そういうことはなかった	o 軽度	o 中等度	o 高度	o 極めて高度

**2. PRO-CTCAE™ Symptom Term:** Difficulty swallowing				
食べ物が飲み込みにくい				
a. この７日の間で、食べ物が飲み込みにくいことは一番ひどい時でどの程度でしたか？				
o そういうことはなかった	o 軽度	o 中等度	o 高度	o 極めて高度

**3. PRO-CTCAE™ Symptom Term:** Mouth/throat sores				
口の中や喉の痛み				
a. この７日の間で、口の中や喉の痛みは一番ひどい時でどの程度でしたか？				
o そういうことはなかった	o 軽度	o 中等度	o 高度	o 極めて高度
b. この７日の間に、口の中や喉の痛みはどの程度ふだんの生活の妨げになりましたか？				
o 全然ならなかった	o 少し	o ある程度	o かなり	o ものすごく

**4. PRO-CTCAE™ Symptom Term:** Cracking at the corners of the mouth (cheilosis/cheilitis)				
口の端のひび割れ				
a. この７日の間で、口の端のひび割れは一番ひどい時でどの程度でしたか？				
o そういうことはなかった	o 軽度	o 中等度	o 高度	o 極めて高度

The PRO-CTCAE™ items and information herein were developed by the Division of Cancer Control and Population Sciences in the NATIONAL CANCER INSTITUTE at the NATIONAL INSTITUTES OF HEALTH, in Bethesda, Maryland, U.S.A. Use of the PRO- CTCAE™ is subject to NCI's Terms of Use.

**5. PRO-CTCAE™ Symptom Term:** Voice quality changes	
声の変化	
a. この７日の間で、声の変化がありましたか？	
o はい	o いいえ

**6. PRO-CTCAE™ Symptom Term:** Hoarseness				
かすれ声				
a. この７日の間で、かすれ声は一番ひどい時でどの程度でしたか？				
o そういうことはなかった	o 軽度	o 中等度	o 高度	o 極めて高度

**7. PRO-CTCAE™ Symptom Term:** Taste changes				
食べ物や飲み物の味がわからない（または、味が変わった）				
a. この７日の間で、食べ物や飲み物の味がわからない（または、味が変わった）ということは一番ひどい時でどの程度でしたか？				
o そういうことはなかった	o 軽度	o 中等度	o 高度	o 極めて高度

**8. PRO-CTCAE™ Symptom Term:** Decreased appetite				
食欲不振				
a. この７日の間で、食欲不振は一番ひどい時でどの程度でしたか？				
o そういうことはなかった	o 軽度	o 中等度	o 高度	o 極めて高度
b. この７日間に、食欲不振はどの程度ふだんの生活の妨げになりましたか？				
o 全然ならなかった	o 少し	o ある程度	o かなり	o ものすごく

**9. PRO-CTCAE™ Symptom Term:** Nausea				
吐き気				
a. この７日の間で、吐き気はありましたか？				
o なかった	o ほとんどなかった	o ときどき	o 頻繁に	o ほとんどいつも
b. この７日の間で、吐き気は一番ひどい時でどの程度でしたか？				
o そういうことはなかった	o 軽度	o 中等度	o 高度	o 極めて高度

The PRO-CTCAE™ items and information herein were developed by the Division of Cancer Control and Population Sciences in the NATIONAL CANCER INSTITUTE at the NATIONAL INSTITUTES OF HEALTH, in Bethesda, Maryland, U.S.A. Use of the PRO- CTCAE™ is subject to NCI's Terms of Use.

**10. PRO-CTCAE™ Symptom Term:** Vomiting				
**嘔吐**				
a. この７日の間に、嘔吐はありましたか？				
o なかった	o ほとんどなかった	o ときどき	o 頻繁に	o ほとんどいつも
b. この７日の間で、嘔吐は一番ひどい時でどの程度でしたか？				
o そういうことはなかった	o 軽度	o 中等度	o 高度	o 極めて高度

**11. PRO-CTCAE™ Symptom Term:** Heartburn				
**胸焼け**				
a. この７日の間で、胸焼けがしましたか？				
o なかった	o ほとんどなかった	o ときどき	o 頻繁に	o ほとんどいつも
b. この７日の間で、胸焼けは一番ひどい時でどの程度でしたか？				
o そういうことはなかった	o 軽度	o 中等度	o 高度	o 極めて高度

**12. PRO-CTCAE™ Symptom Term:** Gas	
**おなら（またはお腹にガスが溜まっている感じ）**	
a. この７日の間で、おならがでる（またはお腹にガスが溜まっている感じがする）回数が増えましたか？	
o はい	o いいえ

**13. PRO-CTCAE™ Symptom Term:** Bloating				
**腹部膨満感（お腹がはった感じ）**				
a. この７日の間に、腹部膨満感（お腹がはった感じ）はありましたか？				
o なかった	o ほとんどなかった	o ときどき	o 頻繁に	o ほとんどいつも
b. この７日の間で、腹部膨満感（お腹がはった感じ）は一番ひどい時でどの程度でしたか？				
o そういうことはなかった	o 軽度	o 中等度	o 高度	o 極めて高度

**14. PRO-CTCAE™ Symptom Term:** Hiccups				
しゃっくり				
a. この７日の間で、しゃっくりがでましたか？				
o なかった	o ほとんどなかった	o ときどき	o 頻繁に	o ほとんどいつも
b. この７日の間で、しゃっくりは一番ひどい時でどの程度でしたか？				
o そういうことはなかった	o 軽度	o 中等度	o 高度	o 極めて高度

**15. PRO-CTCAE™ Symptom Term:** Constipation				
便秘				
a. この７日の間で、便秘は一番ひどい時でどの程度でしたか？				
o そういうことはなかった	o 軽度	o 中等度	o 高度	o 極めて高度

**16. PRO-CTCAE™ Symptom Term:** Diarrhea				
下痢をすること（ゆるい便や水っぽい便）				
a. この７日の間で、下痢をすること（ゆるい便や水っぽい便）がありましたか？				
o なかった	o ほとんどなかった	o ときどき	o 頻繁に	o ほとんどいつも

**17. PRO-CTCAE™ Symptom Term:** Abdominal pain				
腹痛				
a. この７日の間で、腹痛はありましたか？				
o なかった	o ほとんどなかった	o ときどき	o 頻繁に	o ほとんどいつも
b. この７日の間で、腹痛は一番ひどい時でどの程度でしたか？				
o そういうことはなかった	o 軽度	o 中等度	o 高度	o 極めて高度
c. この７日の間に、腹痛はどの程度ふだんの生活の妨げになりましたか？				
o 全然ならなかった	o 少し	o ある程度	o かなり	o ものすごく

The PRO-CTCAE™ items and information herein were developed by the Division of Cancer Control and Population Sciences in the NATIONAL CANCER INSTITUTE at the NATIONAL INSTITUTES OF HEALTH, in Bethesda, Maryland, U.S.A. Use of the PRO- CTCAE™ is subject to NCI's Terms of Use.

**18. PRO-CTCAE™ Symptom Term:** Fecal incontinence				
**便失禁（便通をコントロールできず、もらしてしまう）**				
a. この７日の間で、便失禁（便通をコントロールできず、もらしてしまう）がありましたか？				
o なかった	o ほとんどなかった	o ときどき	o 頻繁に	o ほとんどいつも
b. この７日の間に、便失禁（便通をコントロールできず、もらしてしまう）はどの程度ふだんの生活の妨げになりましたか？				
o 全然ならなかった	o 少し	o ある程度	o かなり	o ものすごく

**19. PRO-CTCAE™ Symptom Term:** Shortness of breath				
**息切れ**				
a. この７日の間で、息切れは一番ひどい時でどの程度でしたか？				
o そういうことはなかった	o 軽度	o 中等度	o 高度	o 極めて高度
b. この７日の間に、息切れはどの程度ふだんの生活の妨げになりましたか？				
o 全然ならなかった	o 少し	o ある程度	o かなり	o ものすごく

**20. PRO-CTCAE™ Symptom Term:** Cough				
**咳（せき）**				
a. この７日の間で、咳（せき）は一番ひどい時でどの程度でしたか？				
o そういうことはなかった	o 軽度	o 中等度	o 高度	o 極めて高度
b. この７日の間に、咳（せき）はどの程度ふだんの生活の妨げになりましたか？				
o 全然ならなかった	o 少し	o ある程度	o かなり	o ものすごく

**21. PRO-CTCAE™ Symptom Term:** Wheezing				
**喘鳴（息をすると胸で笛が鳴るような音がする）**				
a. この７日の間で、喘鳴（息をすると胸で笛が鳴るような音がする）は一番ひどい時でどの程度でしたか？				
o そういうことはなかった	o 軽度	o 中等度	o 高度	o 極めて高度

**22. PRO-CTCAE™ Symptom Term:** Swelling				
**腕や脚のむくみ**				
a. この７日の間に、腕や脚がむくみましたか？				
o むくみはなかった	o ほとんどなかった	o ときどき	o 頻繁に	o ほとんどいつも
b. この７日の間で、腕や脚のむくみは一番ひどい時でどの程度でしたか？				
o そういうことはなかった	o 軽度	o 中等度	o 高度	o 極めて高度
c. この７日の間に、腕や脚のむくみはどの程度ふだんの生活の妨げになりましたか？				
o 全然ならなかった	o 少し	o ある程度	o かなり	o ものすごく

**23. PRO-CTCAE™ Symptom Term:** Heart palpitations				
**心臓がどきどきしたり、鼓動が速くなること（動悸）**				
a. この７日の間で、心臓がどきどきしたり、鼓動が速くなること（動悸）がありましたか？				
o しなかった	o ほとんどしなかった	o ときどき	o 頻繁に	o ほとんどいつも
b. この７日の間で、心臓がどきどきしたり、鼓動が速くなること（動悸）は、一番ひどい時でどの程度でしたか？				
o そういうことはなかった	o 軽度	o 中等度	o 高度	o 極めて高度

**24. PRO-CTCAE™ Symptom Term:** Rash	
**発疹**	
a. この７日の間で、肌に発疹ができましたか？	
o はい	o いいえ

**25. PRO-CTCAE™ Symptom Term:** Skin dryness				
**肌の乾燥**				
a. この７日の間で、肌の乾燥は一番ひどい時でどの程度でしたか？				
o そういうことはなかった	o 軽度	o 中等度	o 高度	o 極めて高度

**26. PRO-CTCAE™ Symptom Term:** Acne				
**顔や胸にできたニキビや吹き出物**				
a. この７日の間で、顔や胸にできたニキビや吹き出物は一番ひどい時でどの程度でしたか？				
o そういうことはなかった	o 軽度	o 中等度	o 高度	o 極めて高度

The PRO-CTCAE™ items and information herein were developed by the Division of Cancer Control and Population Sciences in the NATIONAL CANCER INSTITUTE at the NATIONAL INSTITUTES OF HEALTH, in Bethesda, Maryland, U.S.A. Use of the PRO- CTCAE™ is subject to NCI's Terms of Use.

NCI- PRO-CTCAE™ ITEMS - JAPANESE

Item Library Version 1.0

**27. PRO-CTCAE™ Symptom Term:** Hair loss				
**毛髪が抜ける**				
a. この７日の間で、普段より毛髪が抜けましたか？				
o いいえ	o 少し	o ある程度	o かなり	o ものすごく

**28. PRO-CTCAE™ Symptom Term:** Itching				
**皮膚の痒み**				
a. この７日の間で、皮膚の痒みは一番ひどかった時でどの程度でしたか？				
o そういうことはなかった	o 軽度	o 中等度	o 高度	o 極めて高度

**29. PRO-CTCAE™ Symptom Term:** Hives	
**じんましん**	
a. この７日の間で、じんましん（皮膚が赤く盛り上がって痒い）がでましたか？	
o はい	o いいえ

**30. PRO-CTCAE™ Symptom Term:** Hand-foot syndrome				
**手足症候群（手足にできる皮疹で、ひび割れ、皮がむける、赤くなる、痛みなどの症状）**				
a. この７日の間で、手足症候群（手足にできる皮疹で、ひび割れ、皮がむける、赤くなる、痛みなどの症状）は一番ひどい時でどの程度でしたか？				
o そういうことはなかった	o 軽度	o 中等度	o 高度	o 極めて高度

**31. PRO-CTCAE™ Symptom Term:** Nail loss	
**手足の爪が剥がれる**	
a. この７日の間で、手足の爪が剥がれましたか？	
o はい	o いいえ

**32. PRO-CTCAE™ Symptom Term:** Nail ridging	
**手足の爪の線状、またはデコボコの隆起**	
a. この７日の間で、手足の爪に縦か横に、線状、またはデコボコの隆起ができましたか？	
o はい	o いいえ

The PRO-CTCAE™ items and information herein were developed by the Division of Cancer Control and Population Sciences in the NATIONAL CANCER INSTITUTE at the NATIONAL INSTITUTES OF HEALTH, in Bethesda, Maryland, U.S.A. Use of the PRO- CTCAE™ is subject to NCI’s Terms of Use.

**33. PRO-CTCAE™ Symptom Term:** Nail discoloration	
**爪変色**	
a. この７日の間に、手足の爪に変色がみられましたか？	
o はい	o いいえ

**34. PRO-CTCAE™ Symptom Term:** Sensitivity to sunlight	
**皮膚の日光過敏**	
a. この７日の間で、皮膚が日光に過敏になることがありましたか？	
o はい	o いいえ

**35. PRO-CTCAE™ Symptom Term:** Bed/pressure sores	
**とこずれ**	
a. この７日の間に、とこずれはありましたか？	
o はい	o いいえ

**36. PRO-CTCAE™ Symptom Term:** Radiation skin reaction					
**放射線治療による日焼けや、やけどのような症状は−この質問は放射線治療を受けた方への質問です。受けていない方は、最後の「私には当てはまらない質問です」にチェックしてください。**					
a. この７日の間で、放射線治療による日焼けや、やけどのような症状は一番ひどい時でどの程度でしたか？					
o そういうことはなかった	o 軽度	o 中等度	o 高度	o 極めて高度	o 私には当てはまらない質問です

**37. PRO-CTCAE™ Symptom Term:** Skin darkening	
**皮膚の異常な黒ずみ**	
a. この７日の間で、皮膚が異常に黒ずみましたか？	
o はい	o いいえ

The PRO-CTCAE™ items and information herein were developed by the Division of Cancer Control and Population Sciences in the NATIONAL CANCER INSTITUTE at the NATIONAL INSTITUTES OF HEALTH, in Bethesda, Maryland, U.S.A. Use of the PRO- CTCAE™ is subject to NCI's Terms of Use.

**38. PRO-CTCAE™ Symptom Term:** Stretch marks	
妊娠線のような線	
a. この７日の間に、肌がひきつれたところに妊娠線のような線がでましたか？	
o はい	o いいえ

**39. PRO-CTCAE™ Symptom Term:** Numbness & tingling				
手足の痺れやピリピリ感				
a. この７日の間で、手や足の痺れやピリピリ感は一番ひどい時でどの程度でしたか？				
o そういうことはなかった	o 軽度	o 中等度	o 高度	o 極めて高度
b. この７日の間に、手や足の痺れやピリピリ感はどの程度ふだんの生活の妨げになりましたか？				
o 全然ならなかった	o 少し	o ある程度	o かなり	o ものすごく

**40. PRO-CTCAE™ Symptom Term:** Dizziness				
めまい				
a. この７日の間で、めまいは一番ひどい時でどの程度でしたか？				
o そういうことはなかった	o 軽度	o 中等度	o 高度	o 極めて高度
b. この７日の間に、めまいはどの程度ふだんの生活の妨げになりましたか？				
o 全然ならなかった	o 少し	o ある程度	o かなり	o ものすごく

**41. PRO-CTCAE™ Symptom Term:** Blurred vision				
かすみ目				
a. この７日の間で、目のかすみは一番ひどい時でどの程度でしたか？				
o そういうことはなかった	o 軽度	o 中等度	o 高度	o 極めて高度
b. この７日の間に、目のかすみはどの程度ふだんの生活の妨げになりましたか？				
o 全然ならなかった	o 少し	o ある程度	o かなり	o ものすごく

**42. PRO-CTCAE™ Symptom Term:** Flashing lights	
閃光（稲妻のような光）が走る	
a. この７日の間で、目の前に急に閃光（稲妻のような光）が走ることはありましたか？	
o はい	o いいえ

The PRO-CTCAE™ items and information herein were developed by the Division of Cancer Control and Population Sciences in the NATIONAL CANCER INSTITUTE at the NATIONAL INSTITUTES OF HEALTH, in Bethesda, Maryland, U.S.A. Use of the PRO- CTCAE™ is subject to NCI's Terms of Use.

**43. PRO-CTCAE™ Symptom Term:** Visual floaters	
**目の前に飛ぶ点や線（飛蚊症）**	
a. この７日の間で、目の前に点や線がひらひらと飛んでいる（飛蚊症）のが見えましたか？	
o はい	o いいえ

**44. PRO-CTCAE™ Symptom Term:** Watery eyes				
**涙目（涙が出る）**				
a. この７日の間で、涙目（涙がでる）は一番ひどい時でどの程度でしたか？				
o そういうことはなかった	o 軽度	o 中等度	o 高度	o 極めて高度
b. この７日の間に、涙目（涙がでる）はどの程度普段の生活の妨げになりましたか？				
o 全然ならなかった	o 少し	o ある程度	o かなり	o ものすごく

**45. PRO-CTCAE™ Symptom Term:** Ringing in ears				
**耳鳴り**				
a. この７日の間で、耳鳴りは一番ひどい時でどの程度でしたか？				
o そういうことはなかった	o 軽度	o 中等度	o 高度	o 極めて高度

**46. PRO-CTCAE™ Symptom Term:** Concentration				
**集中力の低下**				
a. この７日の間で、集中力の低下は一番ひどい時でどの程度でしたか？				
o そういうことはなかった	o 軽度	o 中等度	o 高度	o 極めて高度
b. この７日の間に、集中力の低下はどの程度ふだんの生活の妨げになりましたか？				
o 全然ならなかった	o 少し	o ある程度	o かなり	o ものすごく

**47. PRO-CTCAE™ Symptom Term:** Memory				
**物忘れ**				
a. この７日の間で、物忘れは一番ひどい時でどの程度でしたか？				
o そういうことはなかった	o 軽度	o 中等度	o 高度	o 極めて高度
b. この７日の間に、物忘れはどの程度ふだんの生活の妨げになりましたか？				
o 全然ならなかった	o 少し	o ある程度	o かなり	o ものすごく

The PRO-CTCAE™ items and information herein were developed by the Division of Cancer Control and Population Sciences in the NATIONAL CANCER INSTITUTE at the NATIONAL INSTITUTES OF HEALTH, in Bethesda, Maryland, U.S.A. Use of the PRO- CTCAE™ is subject to NCI's Terms of Use.

**48. PRO-CTCAE™ Symptom Term:** General pain				
**痛み**				
a. この７日の間に、体のどこかに痛みはありましたか？				
O なかった	O ほとんどなかった	O ときどき	O 頻繁に	O ほとんどいつも
b. この７日の間で、痛みは一番ひどい時でどの程度でしたか？				
O そういうことはなかった	O 軽度	O 中等度	O 高度	O 極めて高度
c. この７日の間に、痛みはどの程度ふだんの生活の妨げになりましたか？				
O 全然ならなかった	O 少し	O ある程度	O かなり	O ものすごく

**49. PRO-CTCAE™ Symptom Term:** Headache				
**頭痛**				
a. この７日の間で、頭痛はありましたか？				
O なかった	O ほとんどなかった	O ときどき	O 頻繁に	O ほとんどいつも
b. この７日の間で、頭痛は一番ひどい時でどの程度でしたか？				
O そういうことはなかった	O 軽度	O 中等度	O 高度	O 極めて高度
c. この７日の間に、頭痛はどの程度ふだんの生活の妨げになりましたか？				
O 全然ならなかった	O 少し	O ある程度	O かなり	O ものすごく

**50. PRO-CTCAE™ Symptom Term:** Muscle pain				
**筋肉の痛み**				
a. この７日の間に、筋肉の痛みはありましたか？				
O なかった	O ほとんどなかった	O ときどき	O 頻繁に	O ほとんどいつも
b. この７日の間で、筋肉の痛みは一番ひどい時でどの程度でしたか？				
O そういうことはなかった	O 軽度	O 中等度	O 高度	O 極めて高度
c. この７日の間に、筋肉の痛みはどの程度ふだんの生活の妨げになりましたか？				
O 全然ならなかった	O 少し	O ある程度	O かなり	O ものすごく

The PRO-CTCAE™ items and information herein were developed by the Division of Cancer Control and Population Sciences in the NATIONAL CANCER INSTITUTE at the NATIONAL INSTITUTES OF HEALTH, in Bethesda, Maryland, U.S.A. Use of the PRO- CTCAE™ is subject to NCI's Terms of Use.

**51. PRO-CTCAE™ Symptom Term:** Joint pain				
**関節の痛み（肘、膝、肩などの関節）**				
a. この７日の間に、関節（肘、膝、肩などの関節）の痛みはありましたか？				
o なかった	o ほとんどなかった	o ときどき	o 頻繁に	o ほとんどいつも
b. この７日の間で、関節（肘、膝、肩などの関節）の痛みは一番ひどい時でどの程度でしたか？				
o そういうことはなかった	o 軽度	o 中等度	o 高度	o 極めて高度
c. この７日の間に、関節（肘、膝、肩などの関節）の痛みはどの程度ふだんの生活の妨げになりましたか？				
o 全然ならなかった	o 少し	o ある程度	o かなり	o ものすごく

**52. PRO-CTCAE™ Symptom Term:** Insomnia				
**不眠（寝付きが悪い、途中で目が覚める、早く起きてしまう）**				
a. この７日の間で、不眠（寝付きが悪い、途中で目が覚める、早く起きてしまう）は一番ひどい時でどの程度でしたか？				
o そういうことはなかった	o 軽度	o 中等度	o 高度	o 極めて高度
b. この７日の間に、不眠（寝付きが悪い、途中で目が覚める、早く起きてしまう）はどの程度ふだんの生活の妨げになりましたか？				
o 全然ならなかった	o 少し	o ある程度	o かなり	o ものすごく

**53. PRO-CTCAE™ Symptom Term:** Fatigue				
**疲れ、だるさ、活力低下**				
a. この７日の間で、疲れ、だるさ、活力低下は一番ひどい時でどの程度でしたか？				
o そういうことはなかった	o 軽度	o 中等度	o 高度	o 極めて高度
b. この７日の間に、疲れ、だるさ、活力低下はどの程度ふだんの生活の妨げになりましたか？				
o 全然ならなかった	o 少し	o ある程度	o かなり	o ものすごく

The PRO-CTCAE™ items and information herein were developed by the Division of Cancer Control and Population Sciences in the NATIONAL CANCER INSTITUTE at the NATIONAL INSTITUTES OF HEALTH, in Bethesda, Maryland, U.S.A. Use of the PRO- CTCAE™ is subject to NCI’s Terms of Use.

**54. PRO-CTCAE™ Symptom Term:** Anxious				
**不安感**				
a. この７日の間に、不安を感じましたか？				
o 感じなかった	o ほとんど感じなかった	o ときどき	o 頻繁に	o ほとんどいつも
b. この７日の間で、不安が最も強かったときで、どの程度でしたか？				
o そういうことはなかった	o 軽度	o 中等度	o 高度	o 極めて高度
c. この７日の間に、不安を感じることはどの程度ふだんの生活の妨げになりましたか？				
o 全然ならなかった	o 少し	o ある程度	o かなり	o ものすごく

**55. PRO-CTCAE™ Symptom Term:** Discouraged				
**何をしても気分が晴れない**				
a. この７日の間で、何をしても気分が晴れないと感じることはありましたか？				
o なかった	o ほとんどなかった	o ときどき	o 頻繁に	o ほとんどいつも
b. この７日の間で、何をしても気分が晴れないと感じることは一番ひどい時でどの程度でしたか？				
o そういうことはなかった	o 軽度	o 中等度	o 高度	o 極めて高度
c. この７日の間に、何をしても気分が晴れないと感じることはどの程度ふだんの生活の妨げになりましたか？				
o 全然ならなかった	o 少し	o ある程度	o かなり	o ものすごく

**56. PRO-CTCAE™ Symptom Term:** Sad				
**悲しい、不幸だと感じる**				
a. この７日の間に、悲しい、不幸だと感じたことはありましたか？				
o なかった	o ほとんどなかった	o ときどき	o 頻繁に	o ほとんどいつも
b. この７日の間で、一番強く悲しい、不幸だと感じたとき、それはどの程度でしたか？				
o そういうことはなかった	o 軽度	o 中等度	o 高度	o 極めて高度
c. この７日の間に、悲しい、不幸だという気持ちはどの程度ふだんの生活の妨げになりましたか？				
o 全然ならなかった	o 少し	o ある程度	o かなり	o ものすごく

The PRO-CTCAE™ items and information herein were developed by the Division of Cancer Control and Population Sciences in the NATIONAL CANCER INSTITUTE at the NATIONAL INSTITUTES OF HEALTH, in Bethesda, Maryland, U.S.A. Use of the PRO- CTCAE™ is subject to NCI's Terms of Use.

**57. PRO-CTCAE™ Symptom Term:** Irregular periods/vaginal bleeding		
生理不順		
a. この７日の間で、生理不順がありましたか？		
o あった	o なかった	o 私には当てはまらない質問です

**58. PRO-CTCAE™ Symptom Term:** Missed expected menstrual period		
予定された生理がこない		
a. この７日の間で、予定された生理がこなかったですか？		
o はい	o いいえ	o 私にはあてはまらない質問です

**59. PRO-CTCAE™ Symptom Term:** Vaginal discharge				
普段と異なるおりもの				
a. この７日の間に、膣から普段と異なるおりものがありましたか？				
o いいえ	o 少し	o ある程度	o かなり	o ものすごく

**60. PRO-CTCAE™ Symptom Term:** Vaginal dryness				
膣の乾燥				
a. この７日の間で、膣の乾燥は一番ひどい時でどの程度でしたか？				
o そういうことはなかった	o 軽度	o 中等度	o 高度	o 極めて高度

**61. PRO-CTCAE™ Symptom Term:** Painful urination				
排尿時の痛みや焼けるような感じ				
a. この７日の間で、排尿時の痛みや焼けるような感じは一番ひどい時でどの程度でしたか？				
o そういうことはなかった	o 軽度	o 中等度	o 高度	o 極めて高度

The PRO-CTCAE™ items and information herein were developed by the Division of Cancer Control and Population Sciences in the NATIONAL CANCER INSTITUTE at the NATIONAL INSTITUTES OF HEALTH, in Bethesda, Maryland, U.S.A. Use of the PRO- CTCAE™ is subject to NCI's Terms of Use.

**62. PRO-CTCAE™ Symptom Term:** Urinary urgency				
**切迫した尿意**				
a. この７日の間で、突然切迫した尿意を感じたことはありましたか？				
O なかった	O ほとんどなかった	O ときどき	O 頻繁に	O ほとんどいつも
b. この７日の間に、突然の切迫した尿意はどの程度ふだんの生活の妨げになりましたか？				
O 全然ならなかった	O 少し	O ある程度	O かなり	O ものすごく

**63. PRO-CTCAE™ Symptom Term:** Urinary frequency				
**尿が近い**				
a. この７日の間で、尿が近いことはありましたか？				
O なかった	O ほとんどなかった	O ときどき	O 頻繁に	O ほとんどいつも
b. この７日の間に、尿が近いことはどの程度ふだんの生活の妨げになりましたか？				
O 全然ならなかった	O 少し	O ある程度	O かなり	O ものすごく

**64. PRO-CTCAE™ Symptom Term:** Change in usual urine color	
**尿の色が普段と違う**	
a. この７日の間で、尿の色が普段と違うことがありましたか？	
O はい	O いいえ

**65. PRO-CTCAE™ Symptom Term:** Urinary incontinence				
**尿失禁（または尿漏れ）**				
a. この７日の間で、尿失禁（または尿漏れ）したことがありましたか？				
O なかった	O ほとんどなかった	O ときどき	O 頻繁に	O ほとんどいつも
b. この７日の間に、尿失禁（または尿漏れ）はどの程度ふだんの生活の妨げになりましたか？				
O 全然ならなかった	O 少し	O ある程度	O かなり	O ものすごく

The PRO-CTCAE™ items and information herein were developed by the Division of Cancer Control and Population Sciences in the NATIONAL CANCER INSTITUTE at the NATIONAL INSTITUTES OF HEALTH, in Bethesda, Maryland, U.S.A. Use of the PRO- CTCAE™ is subject to NCI's Terms of Use.

**66. PRO-CTCAE™ Symptom Term:** Achieve and maintain erection						
**勃起しにくい、またはすぐ萎える**						
a. この７日の間で、勃起しにくい、またはすぐ萎えるということは一番ひどい時でどの程度でしたか？						
o そういうことはなかった	o 軽度	o 中等度	o 高度	o 極めて高度	o 性行為はしていない	o 回答辞退

**67. PRO-CTCAE™ Symptom Term:** Ejaculation						
**射精に関する問題**						
a. この７日の間で、射精に関する問題がありましたか？						
o なかった	o ほとんどなかった	o ときどき	o 頻繁に	o ほとんどいつも	o 性行為はしていない	o 回答辞退

**68. PRO-CTCAE™ Symptom Term:** Decreased libido						
**性欲減退**						
a. この７日の間で、性欲減退は一番ひどい時でどの程度でしたか？						
o そういうことはなかった	o 軽度	o 中等度	o 高度	o 極めて高度	o 性行為はしていない	o 回答辞退

**69. PRO-CTCAE™ Symptom Term:** Delayed orgasm			
**オルガズムやクライマックスまでに時間がかかりすぎる**			
a. この７日の間に、普段に比べてオルガズムやクライマックスまでに時間がかかりすぎると感じたことはありますか？			
o はい	o いいえ	o 性行為はしていない	o 回答辞退

**70. PRO-CTCAE™ Symptom Term:** Unable to have orgasm			
**普段に比べてオルガズムやクライマックスが無い**			
a. この７日の間で、セックスをしてもオルガズムやクライマックスが無いことがありましたか？			
o はい	o いいえ	o 性行為はしていない	o 回答辞退

The PRO-CTCAE™ items and information herein were developed by the Division of Cancer Control and Population Sciences in the NATIONAL CANCER INSTITUTE at the NATIONAL INSTITUTES OF HEALTH, in Bethesda, Maryland, U.S.A. Use of the PRO- CTCAE™ is subject to NCI’s Terms of Use.

**71. PRO-CTCAE™ Symptom Term:** Pain w/sexual intercourse						
性交時の痛み（膣内）						
a. この７日の間で、性交時の痛み（膣内）は一番ひどい時でどの程度でしたか？						
o そういうことはなかった	o 軽度	o 中等度	o 高度	o 極めて高度	o 性行為はしていない	o 回答辞退

**72. PRO-CTCAE™ Symptom Term:** Breast swelling and tenderness				
乳房の張りや圧痛				
a. この７日の間で、乳房の張りや圧痛（触ったり押したりすると痛い）は一番ひどい時でどの程度でしたか？				
o そういうことはなかった	o 軽度	o 中等度	o 高度	o 極めて高度

**73. PRO-CTCAE™ Symptom Term:** Bruising	
内出血（黒や青のあざ）	
a. この７日の間に、よく内出血（黒や青のあざ）しましたか？	
o はい	o いいえ

**74. PRO-CTCAE™ Symptom Term:** Chills				
震えや寒け				
a. この７日の間に、震えや寒けを感じましたか？				
o 感じなかった	o ほとんど感じなかった	o ときどき	o 頻繁に	o ほとんどいつも
b. この７日の間で、震えや寒けは一番ひどい時でどの程度でしたか？				
o そういうことはなかった	o 軽度	o 中等度	o 高度	o 極めて高度

The PRO-CTCAE™ items and information herein were developed by the Division of Cancer Control and Population Sciences in the NATIONAL CANCER INSTITUTE at the NATIONAL INSTITUTES OF HEALTH, in Bethesda, Maryland, U.S.A. Use of the PRO- CTCAE™ is subject to NCI's Terms of Use.

**75. PRO-CTCAE™ Symptom Term:** Increased sweating				
**昼間または夜間の予想外、または大量の汗**				
a. この７日の間に、昼間または夜、（体のほてりやのぼせとは関係なく）予想外に汗をかく、または、大量の汗をかくことがありましたか？				
o なかった	o ほとんどなかった	o ときどき	o 頻繁に	o ほとんどいつも
b. この７日の間で、昼間または夜にかいた、（体のほてりやのぼせとは関係なく）予想外の汗、または大量の汗は一番ひどい時でどの程度でしたか？				
o そういうことはなかった	o 軽度	o 中等度	o 高度	o 極めて高度

**76. PRO-CTCAE™ Symptom Term:** Decreased sweating	
**汗が予想外に少ない**	
a. この７日の間で、汗が予想外に少ないということはありましたか？	
o はい	o いいえ

**77. PRO-CTCAE™ Symptom Term:** Hot flashes				
**ほてりやのぼせ**				
a. この７日の間で、体のほてりやのぼせを感じたことはありましたか？				
o なかった	o ほとんどなかった	o ときどき	o 頻繁に	o ほとんどいつも
b. この７日の間で、体のほてりやのぼせは一番ひどい時でどの程度でしたか？				
o そういうことはなかった	o 軽度	o 中等度	o 高度	o 極めて高度

**78. PRO-CTCAE™ Symptom Term:** Nosebleed				
**鼻血**				
a. この７日の間で、鼻血がでましたか？				
o でなかった	o ほとんどでなかった	o ときどき	o 頻繁に	o ほとんどいつも
b. この７日の間で、鼻血は一番ひどい時でどの程度でしたか？				
o そういうことはなかった	o 軽度	o 中等度	o 高度	o 極めて高度

The PRO-CTCAE™ items and information herein were developed by the Division of Cancer Control and Population Sciences in the NATIONAL CANCER INSTITUTE at the NATIONAL INSTITUTES OF HEALTH, in Bethesda, Maryland, U.S.A. Use of the PRO- CTCAE™ is subject to NCI's Terms of Use.

**79. PRO-CTCAE™ Symptom Term:** Pain and swelling at injection site		
**注射部が痛む、腫れる、赤くなる**		
a. この７日の間で、注射または点滴をしたところに、痛み、腫れ、赤くなることがありましたか？		
o はい	o いいえ	o 私にはあてはまらない質問です

**80. PRO-CTCAE™ Symptom Term:** Body odor				
**体臭**				
a. この７日の間で、あなたの体臭は一番強い時でどの程度でしたか？				
o そういうことはなかった	o 軽度	o 中等度	o 高度	o 極めて高度

The PRO-CTCAE™ items and information herein were developed by the Division of Cancer Control and Population Sciences in the NATIONAL CANCER INSTITUTE at the NATIONAL INSTITUTES OF HEALTH, in Bethesda, Maryland, U.S.A. Use of the PRO- CTCAE™ is subject to NCI's Terms of Use.

NCI- PRO-CTCAE™ ITEMS - JAPANESE

Item Library Version 1.0

OTHER SYMPTOMS	
その他の症状	
Do you have any other symptoms that you wish to report?<br><br>上記以外で、何か報告したいと思う症状などがありますか？	
O Yes	O No
O はい	O いいえ
**Please list any other symptoms:**<br>**他にも症状がありましたら列記してください。**	
1.	過去７日間で、この症状が最もひどい時に、その程度はどのくらいでしたか？<br>O そういうことはなかったO 軽度O 中等度O 高度O 極めて高度
2.	過去７日間で、この症状が最もひどい時に、その程度はどのくらいでしたか？<br>O そういうことはなかったO 軽度O 中等度O 高度O 極めて高度
3.	過去７日間で、この症状が最もひどい時に、その程度はどのくらいでしたか？<br>O そういうことはなかったO 軽度O 中等度O 高度O 極めて高度
4.	過去７日間で、この症状が最もひどい時に、その程度はどのくらいでしたか？<br>O そういうことはなかったO 軽度O 中等度O 高度O 極めて高度
5.	過去７日間で、この症状が最もひどい時に、その程度はどのくらいでしたか？<br>O そういうことはなかったO 軽度O 中等度O 高度O 極めて高度

The PRO-CTCAE™ items and information herein were developed by the Division of Cancer Control and Population Sciences in the NATIONAL CANCER INSTITUTE at the NATIONAL INSTITUTES OF HEALTH, in Bethesda, Maryland, U.S.A. Use of the PRO- CTCAE™ is subject to NCI's Terms of Use.

NCI- PRO-CTCAE™ ITEMS - JAPANESE

Item Library Version 1.0

The PRO-CTCAE™ items and information herein were developed by the Division of Cancer Control and Population Sciences in the NATIONAL CANCER INSTITUTE at the NATIONAL INSTITUTES OF HEALTH, in Bethesda, Maryland, U.S.A. Use of the PRO- CTCAE™ is subject to NCI's Terms of Use.

Version date: 4/26/2020
