# Supplementary material for: Mitigation of paclitaxel-induced peripheral neuropathy in breast cancer patients using limb-cooling apparatus: a study protocol for a randomized controlled trial
Source: Front Oncol. 2023 Jul 7;13:1216813. doi: 10.3389/fonc.2023.1216813 (PMC10361568; doi:10.3389/fonc.2023.1216813)
Supplement: Supplementary file 1 [file DataSheet_1.zip › CECILIA_PNQ.pdf]

ご本人記入欄

記入日：（西暦）＿＿＿＿＿年＿＿＿＿月＿＿＿＿＿日

【Patient Neurotoxicity Questionnaire (PNQ)】

感覚　該当する☐に✓を記入してください。

☐	☐	☐	☐	☐
A	B	C	D	E
手や足にしびれや痛み、知覚鈍麻を感じない	手や足に軽いしびれや痛み、知覚鈍麻を感じるが、日常生活に支障なし	手や足に中等度のしびれや痛み、知覚鈍麻を感じるが、日常生活に支障なし	手や足にやや強めのしびれや痛み、知覚鈍麻を感じ、日常生活に支障あり	手や足に強いしびれや痛み、知覚鈍麻を感じ、ほとんどの日常生活の妨げになる

運動　該当する☐に✓を記入してください。

☐	☐	☐	☐	☐
A	B	C	D	E
手足に筋力低下（脱力感）を感じない	手足に軽い筋力低下（脱力感）を感じるが、日常生活に支障なし	手足に中等度の筋力低下（脱力感）を感じるが、日常生活に支障なし	手足にやや強めの筋力低下（脱力感）を感じ、日常生活に支障あり	手足に強い筋力低下（脱力感）を感じ、ほとんどの日常生活の妨げになる

☐に✓を記入してどのような活動に支障があるか示してください。

☐洋服のボタンかけ	☐ドアを開く	☐ベルトのバックルを締める	☐字を書く	☐裁縫
☐ナイフを使う	☐コンタクトレンズの着脱	☐眠る	☐歩く	☐仕事
☐フォークを使う	☐（携帯）電話を使う	☐階段を上る	☐アクセサリーを着ける	☐靴紐を結ぶ
☐スプーンを使う	☐リモコン操作	☐キーボードを打つ	☐編み物	☐自動車運転
	☐箸を使う	☐その他（ご記入ください）＿＿＿＿＿＿＿＿＿＿		
