# Supplementary material for: Mitigation of paclitaxel-induced peripheral neuropathy in breast cancer patients using limb-cooling apparatus: a study protocol for a randomized controlled trial
Source: Front Oncol. 2023 Jul 7;13:1216813. doi: 10.3389/fonc.2023.1216813 (PMC10361568; doi:10.3389/fonc.2023.1216813)
Supplement: Supplementary file 1 [file DataSheet_1.zip › CIPN20 Japanese 1.1.pdf]

<u>EORTC QLQ – CINP20</u>

下記のような症状や問題が起こることがあります。項目ごとに、<u>ここ１週間</u>のあなたの状態にもっともよくあてはまる番号をひとつだけ選び、〇で囲んでください。

この一週間について:	まったくない	少しある	多い	とても多い
31. 手や指がジンジン痛みましたか。	1	2	3	4
32. 足やつま先がジンジン痛みましたか。	1	2	3	4
33. 手や指の感覚が麻痺(まひ)しましたか。	1	2	3	4
34. 足やつま先の感覚が麻痺(まひ)しましたか。	1	2	3	4
35. 手や指に鋭い痛みや焼けるような痛みがありましたか。	1	2	3	4
36. 足やつま先に鋭い痛みや焼けるような痛みがありましたか。	1	2	3	4
37. 手の筋肉が<u>つる</u>ことがありましたか。	1	2	3	4
38. 足の筋肉が<u>つる</u>ことがありましたか。	1	2	3	4
39. 踏んだ地面の感覚がはっきりせず、立ったり歩いたりするのに問題がありましたか。	1	2	3	4
40. 水の熱さと冷たさを区別するのが難しかったですか。	1	2	3	4
41. ペン（鉛筆）を握るのが難しく、字が書きにくかったですか。	1	2	3	4
42. 指で小さなものを扱うのが難しかったですか（例えば小さなボタンをかける）。	1	2	3	4
43. 手に力が入らず、ペットボトルなどのふたを開けるのが難しかったですか。	1	2	3	4
44. つま先が持ち上げにくくて、歩くのが難しかったですか。	1	2	3	4

<u>次のページにお進みください。</u>

この一週間について:	まったくない	少しある	多い	とても多い
45. 足に力が入りにくくて、階段を登ったり椅子から立ち上がったりするのが難しかったですか。	1	2	3	4
46. 座った状態や寝た状態から立ち上がる際に頭がフラフラしましたか。	1	2	3	4
47. 目がかすんだりしましたか。	1	2	3	4
48. 耳が聞こえにくかったですか。	1	2	3	4

車の運転をする方のみお答えください:

49. ペダルを踏むのが難しかったですか。	1	2	3	4

男性のみお答えください:

50. 勃起(ぼっき)をさせたり勃起を持続させたりするのが難しかったですか。	1	2	3	4

© QLQ-CINP20 Copyright 2003, EORTC Quality of life Group. All rights reserved
