# Supplementary material for: Mitigation of paclitaxel-induced peripheral neuropathy in breast cancer patients using limb-cooling apparatus: a study protocol for a randomized controlled trial
Source: Front Oncol. 2023 Jul 7;13:1216813. doi: 10.3389/fonc.2023.1216813 (PMC10361568; doi:10.3389/fonc.2023.1216813)
Supplement: Supplementary file 1 [file DataSheet_1.zip › QLQ-C30 Japanese 1.4.pdf]

EORTC QLQ-C30 (version 3)

私達は、あなたとあなたの健康状態について関心を持っています。あなたの状態に、もっともよく当てはまる番号一つを○で囲み、全設問にお答え下さい。「正しい」答えや「誤った」答え、といったものはありません。なお、お答え頂いた内容については秘密厳守とさせていただきます。

あなたの名前の頭文字を書いて下さい。　姓：___ 名：___（例：山田花子さん。姓：や　名：は）
あなたの生年月日を書いて下さい。　_____年（明・大・昭・平____年）____月____日生
今日の日付を書いて下さい。　20___年（令和____________年）____月____日

		まったくない	少しある	多い	とても多い
1.	重い買い物袋やスーツケースを運ぶなどの力仕事に支障がありますか。	1	2	3	4
2.	長い距離を歩くことに支障がありますか。	1	2	3	4
3.	屋外の短い距離を歩くことに支障がありますか。	1	2	3	4
4.	一日中ベッドやイスで過ごさなければなりませんか。	1	2	3	4
5.	食べること、衣類を着ること、顔や体を洗うこと、トイレを使うことに人の手を借りる必要がありますか。	1	2	3	4

この一週間について:		まったくない	少しある	多い	とても多い
6.	仕事をすることや日常生活活動に支障がありましたか。	1	2	3	4
7.	趣味やレジャーをするのに支障がありましたか。	1	2	3	4
8.	息切れがありましたか。	1	2	3	4
9.	痛みがありましたか。	1	2	3	4
10.	休息をとる必要がありましたか。	1	2	3	4
11.	睡眠に支障がありましたか。	1	2	3	4
12.	体力が弱くなったと感じましたか。	1	2	3	4
13.	食欲がないと感じましたか。	1	2	3	4
14.	吐き気がありましたか。	1	2	3	4
15.	吐きましたか。	1	2	3	4
16.	便秘がありましたか。	1	2	3	4

次のページにお進みください

この一週間について:	まったく ない	少し ある	多い	とても 多い
17. 下痢がありましたか。	1	2	3	4
18. 疲れていましたか。	1	2	3	4
19. 痛みがあなたの日々の活動のさまたげになりましたか。	1	2	3	4
20. ものごとに集中しにくいことがありましたか。たとえば 新聞を読むときや、テレビを見るとき。	1	2	3	4
21. 緊張した気分でしたか。	1	2	3	4
22. 心配がありましたか。	1	2	3	4
23. 怒りっぽい気分でしたか。	1	2	3	4
24. 落ち込んだ気分でしたか。	1	2	3	4
25. もの覚えが悪くなったと思いましたか。	1	2	3	4
26. 身体の調子や治療の実施が、家族の一員としての あなたの生活のさまたげになりましたか。	1	2	3	4
27. 身体の調子や治療の実施が、あなたの社会的な活動の さまたげになりましたか。	1	2	3	4
28. 身体の調子や治療の実施が、あなたの経済上の 問題になりましたか。	1	2	3	4

次の質問では、1から7の数字のうち、あなたにもっともよく当てはまる数字を○で囲んで下さい。

29. この一週間のあなたの健康状態は全体としてどの程度だったでしょうか。

1	2	3	4	5	6	7
とても悪い						とてもよい

30. この一週間、あなたの全体的な生活の質はどの程度だったでしょうか。

1	2	3	4	5	6	7
とても悪い						とてもよい

QLQ-C30 Copyright 1995 EORTC Quality of Life Group. 無断複写・転載を禁じます. バージョン 3.0
