# Supplementary material for: Mitigation of paclitaxel-induced peripheral neuropathy in breast cancer patients using limb-cooling apparatus: a study protocol for a randomized controlled trial
Source: Front Oncol. 2023 Jul 7;13:1216813. doi: 10.3389/fonc.2023.1216813 (PMC10361568; doi:10.3389/fonc.2023.1216813)
Supplement: Supplementary file 1 [file DataSheet_1.zip › CIPN20 English.doc]

**EORTC QLQ – CIPN20**

Patients sometimes report that they have the following symptoms or problems. Please indicate the extent to which you have experienced these symptoms or problems during the past week. Please answer by circling the number that best applies to you.

**During the past week :	Not at	A	Quite	Very**

**All	Little	a Bit	Much**

31	Did you have tingling fingers or hands?	1	2	3	4

32	Did you have tingling toes or feet?	1	2	3	4

33	Did you have numbness in your fingers or hands?	1	2	3	4

34	Did you have numbness in your toes or feet?	1	2	3	4

35	Did you have shooting or burning pain in your fingers or hands?	1	2	3	4

36	Did you have shooting or burning pain in your toes or feet?	1	2	3	4

37	Did you have cramps in your hands?	1	2	3	4

38	Did you have cramps in your feet?	1	2	3	4

39	Did you have problems standing or walking because
of difficulty feeling the ground under your feet?	1	2	3	4

40	Did you have difficulty distinguishing
between hot and cold water?	1	2	3	4

41	Did you have a problem holding a pen, which
made writing difficult?	1	2	3	4

42	Did you have difficulty manipulating small objects with
your fingers (for example, fastening small buttons)?	1	2	3	4

43	Did you have difficulty opening a jar or
bottle because of weakness in your hands?	1	2	3	4

44	Did you have difficulty walking because your
feet dropped downwards?	1	2	3	4

Please go on to the next page

**During the past week :	Not at	A	Quite	Very**

**All	Little	a Bit	Much**

45	Did you have difficulty climbing stairs or getting up
out of a chair because of weakness in your legs?	1	2	3	4

46	Were you dizzy when standing up from
a sitting or lying position?	1	2	3	4

47	Did you have blurred vision?	1	2	3	4

48	Did you have difficulty hearing?	1	2	3	4

**Please answer the following question only if you drive a car**

49	Did you have difficulty using the pedals?	1	2	3	4

**Please answer the following question only if you are a man**

50	Did you have difficulty getting or maintaining an erection?	1	2	3	4
