# Supplementary material for: Mitigation of paclitaxel-induced peripheral neuropathy in breast cancer patients using limb-cooling apparatus: a study protocol for a randomized controlled trial
Source: Front Oncol. 2023 Jul 7;13:1216813. doi: 10.3389/fonc.2023.1216813 (PMC10361568; doi:10.3389/fonc.2023.1216813)
Supplement: Supplementary file 1 [file DataSheet_1.zip › QLQ-C30 English.doc]

**EORTC QLQ-C30 (version 3)**

We are interested in some things about you and your health. Please answer all of the questions yourself by circling the number that best applies to you. There are no "right" or "wrong" answers. The information that you provide will remain strictly confidential.

Please fill in your initials:	****

Your birthdate (Day, Month, Year):	****

Today's date (Day, Month, Year):	31	****

__________________________________________________________________________________________

#	Not at	A	Quite	Very

#	All	Little	a Bit	Much

1.	Do you have any trouble doing strenuous activities,

like carrying a heavy shopping bag or a suitcase?	1	2	3	4

2.	Do you have any trouble taking a long walk?	1	2	3	4

3.	Do you have any trouble taking a short walk outside of the house?	1	2	3	4

4.	Do you need to stay in bed or a chair during the day?	1	2	3	4

5.	Do you need help with eating, dressing, washing

yourself or using the toilet?	1	2	3	4

# During the past week: Not at A Quite Very

#	All	Little	a Bit	Much

6.	Were you limited in doing either your work or other daily activities?	1	2	3	4

7.	Were you limited in pursuing your hobbies or other

leisure time activities?	1	2	3	4

8.	Were you short of breath?	1	2	3	4

9.	Have you had pain?	1	2	3	4

10.	Did you need to rest?	1	2	3	4

11.	Have you had trouble sleeping?	1	2	3	4

12.	Have you felt weak?	1	2	3	4

13.	Have you lacked appetite?	1	2	3	4

14.	Have you felt nauseated?	1	2	3	4

15.	Have you vomited?	1	2	3	4

16.	Have you been constipated?	1	2	3	4

Please go on to the next page

# During the past week: Not at A Quite Very

#	All	Little	a Bit	Much

17.	Have you had diarrhea?	1	2	3	4

18.	Were you tired?	1	2	3	4

19.	Did pain interfere with your daily activities?	1	2	3	4

20.	Have you had difficulty in concentrating on things,

like reading a newspaper or watching television?	1	2	3	4

21.	Did you feel tense?	1	2	3	4

22.	Did you worry?	1	2	3	4

23.	Did you feel irritable?	1	2	3	4

24.	Did you feel depressed?	1	2	3	4

25.	Have you had difficulty remembering things?	1	2	3	4

26.	Has your physical condition or medical treatment

interfered with your family life?	1	2	3	4

27.	Has your physical condition or medical treatment

interfered with your social activities?	1	2	3	4

28.	Has your physical condition or medical treatment

caused you financial difficulties?	1	2	3	4

For the following questions please circle the number between 1 and 7 that
best applies to you

29.	How would you rate your overall health during the past week?

1	2	3	4	5	6	7

Very poor	Excellent

30.	How would you rate your overall quality of life during the past week?

1	2	3	4	5	6	7

Very poor	Excellent
